# Fast-Track Surgery in Gynaecology and Gynaecologic Oncology: A Review of a Rolling Clinical Audit

**DOI:** 10.5402/2012/368014

**Published:** 2012-12-24

**Authors:** Jonathan Carter

**Affiliations:** ^1^The University of Sydney, Sydney, NSW 2006, Australia; ^2^Sydney Gynaecological Oncology Group, Sydney Cancer Centre, Royal Prince Alfred Hospital, Camperdown, NSW 2050, Australia

## Abstract

Clinical audit is the process by which clinicians are able to demonstrate to themselves, their patients, hospital administrators, and healthcare financial providers the outcome and safety of their clinical practice. It is a process by which the public can be assured of safety and outcomes. A fast-track surgery program was initiated in January 2008, and this paper represents a rolling clinical audit of the outcomes of that program until the end of June 2012. Three hundred and eighty-nine patients underwent fast track surgical management after having a laparotomy for suspected or confirmed gynaecological cancer. There were no exclusions and the data presented represents the practice and outcomes of all patients referred to a single gynaecological oncologist. The majority of patients were deemed to have complex surgical procedures performed usually through a vertical midline incision. One third of patients had a nonzero performance status, median weight was 68 kilograms, and median BMI was 26.5 with 31% being classified as obese. Median operating time was 2.25 hours, and the median estimated blood loss was 175 mL. Overall the median length of stay (LOS) was 3 days with 95% of patients tolerating early oral feeding. Four percent of patients required readmission, and 0.5% were required to return to the operating room. Whilst the wound infection rate was 2.6%, there were no ureteric, bowel or neurovascular injuries. Overall there were 2 bladder injuries (0.5%), and the incidence of venous thromboembolism was 1%. Subset analysis was also undertaken. Whilst a number of variables were associated with reduced LOS, on multivariate analysis, benign pathology, shorter operating time, and the ability to tolerate early oral feeding were found to be significant. The data and experience presented is the largest and most extensive reported in the literature relating to fast-track surgery in gynaecology and gynaecologic oncology. The public can be reassured of the safety and improved outcomes that can be achieved after the introduction of such a program.

## 1. Introduction

Clinical audit is one of the fundamental principles of clinical governance, the process by which clinicians improve the quality of the care they provide. The process involves regularly collecting and measuring activity and outcomes, and analyzing and comparing these outcomes with current or “recognized standards,” together with a rigorous peer review process. It makes clinicians accountable to the public, to constantly monitor and maintain high standards, to be transparent and accountable for those standards, to identify problems and address them, and to constantly improve on those standards to improve overall quality of care. It is what the public expect [1]. The key feature of audit is that it involves reviewing actual and all surgical performance outcomes. It provides powerful information to the consumer (patient) and health care provider (Hospital, LHN, Government) as to the outcomes really achieved in a real life scenario, rather than in an artificial trial environment. In lay terms, the purpose of audit is to confirm that your outcomes are, what you say or think they are [2]. It has been shown quite clearly from cardiac surgery that structured data collection, analysis, and feedback to clinicians improves the quality of outcomes [3].

Fast-track surgery (FTS) programs are also known as Enhanced Recovery after Surgery (ERAS) or Rapid or Accelerated Recovery after Surgery programs. They are not new, nor are they complicated. They were first described by Kehlet in Denmark [4], and the principles have been adopted by most surgical specialties worldwide [5, 6]. The basic tenant of such programs is to enhance recovery after surgery, allowing earlier discharge with improved patient outcomes. Such programs derive their success from being multidisciplinary with all members of the team having an important function. Team members include surgeons, nurses, anaesthetist, pain specialists, ward nursing staff, social worker, occupational, and physical therapy staff [7, 8].

Whilst much of the evidence supporting FTS has been published in the colorectal literature [9, 10], there are increasing reports in the gynaecological literature attesting to the safety and improved outcomes [11–17]; however, prospective randomized controlled studies are lacking [18]. Despite Victorian Department of Health, Cochrane and Australian Safety and Efficacy Register of New Interventional Procedures-Surgical (ASERNIP-S) reviews, the concept and principles have been slow to be adopted in Australia [18, 19].

**Figure 1:**
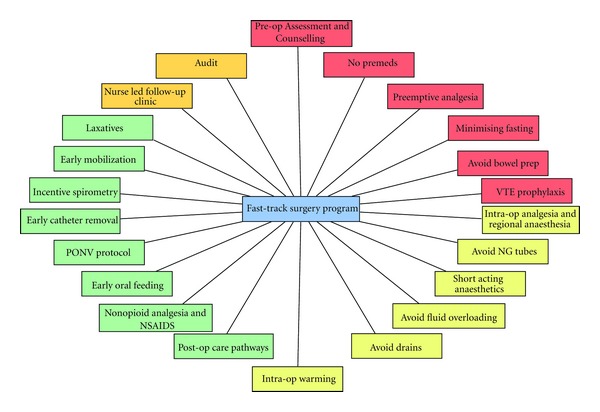
Elements of a fast-track surgery program.

**Figure 2:**
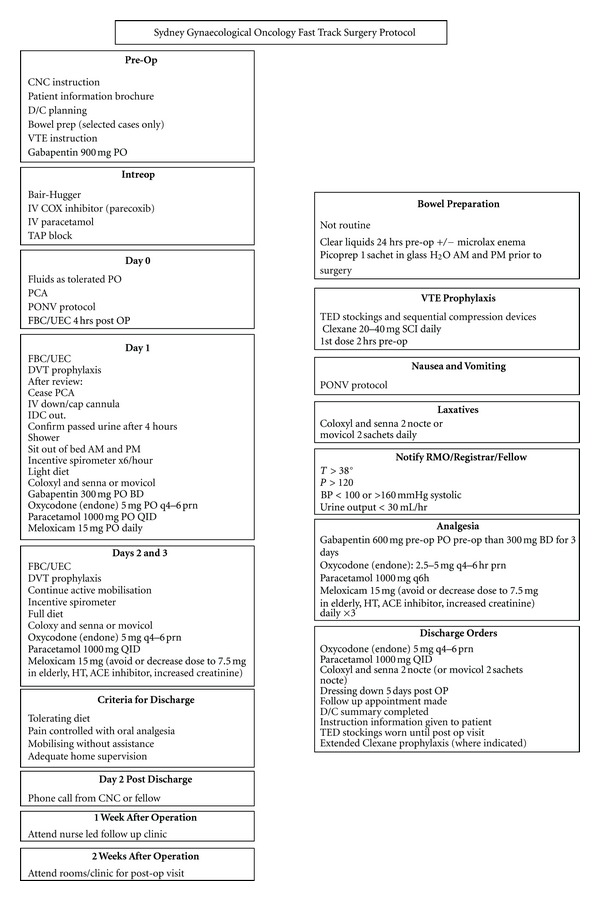
Fast-track surgery program.

FTS programs incorporate a number of elements and are not just clinical pathways (Figures 1 and 2). Many of these elements are already practiced by surgeons, but few embrace the entirety to gain the maximum benefits for the patient. By minimizing stress and maintaining normal physiology as much as possible, the catabolic insults of surgery and anaesthesia can be minimised, optimising patient outcomes and as a consequence reducing length of stay (LOS).

Evidence from other surgical specialties would indicate that the completion of a fast track surgical program, is not only achievable, but is safe, and allows early discharge with a low risk of readmission and improved outcomes [20]. Three blinded studies comparing minimally invasive operations to the conventional open surgical approach (with fast track principles) have shown no obvious clinical advantages to the minimally invasive approach after colonic [21], appendectomy [22], and hip replacement surgery [23]. Furthermore, in a blinded study of laparoscopic cholecystectomy versus small incision cholecystectomy, where bias was eliminated by using identical wound dressings, it was found that the laparoscopic approach took longer to perform and did not have significant advantages in terms of hospital stay or postoperative recovery [24].

Our study provides the largest real, base-line or “recognised standard” on laparotomy patients managed by FTS for the surgical management of complex gynaecology and gynaecological cancer.

## 2. Program Outline

The program commences with preoperative counseling of the patient, optimizing medical care of those with diabetes, cardiovascular, or other comorbidities. Patients are counseled by both admitting surgeon and nurse (in our case a dedicated clinical nurse consultant or nurse practitioner). Issues discussed include the rationale and sequence of the program, informing the patient of their anticipated LOS and the criteria for discharge. That narcotic analgesia would be limited and adequate analgesia provided by a combination of intraoperative parecoxib and transverse abdominis plane (TAP) block [14, 25]. Mechanical bowel preparations are not routine, fluid balance optimized to retain as close to normal intravascular volume and that unnecessary tissue trauma is avoided by good surgical technique. Strict attention to haemostasis is important and drains are avoided. Postoperatively meloxicam 15 mg is prescribed for 3 days with regular paracetamol 1000 mg every 6 hours. Oral liquids are allowed on the night of surgery and light diet on post-op day 1 with rapid progression thereafter. Movicol or Coloxyl with Senna is commenced routinely on post-op day 1 and continued after discharge. All patients receive perioperative enoxaparin sodium 20–40 mg SCI which is continued until discharge. Selected high risk patients are offered extended enoxaparin sodium prophylaxis. Intraoperatively mechanical sequential compression devices are employed and all patients have knee high TED stockings fitted and worn postoperatively for at least 1 month. In addition, all patients receive intravenous ceftriaxone 1 g prior to surgery unless allergic to penicillin or cephalosporin's, in which case clindamycin is usually prescribed. Patients are mobilized on day 1 after surgery and catheters and IV fluids are removed on day 1 if the patient is haemodynamically stable. Patients are given an incentive spirometer or “Triflow” and encouraged to use the device 6 times per hour. Criteria for discharge include the patient adequately mobilizing without assistance, tolerating early oral feeding, managing their pain and discomfort with oral analgesia and having adequate home supervision. Post-discharge patients receive a follow-up phone call from our CNC within 3 days of discharge and attend the nurse led follow up clinic 1 week after discharge.

## 3. Data Collection

This audit reports the experience of 4.5 full years of patients referred to a single gynaecological oncologist, for the surgical management of suspected or confirmed gynaecological malignancy. The audit includes all patients taken to the operating room for the calendar years 2008, 2009, 2010, 2011, and for the first six months of 2012, who underwent a laparotomy. There were no exclusions and no exceptions. Data was collected in a real time fashion on the author's personal database and analysis undertaken in a retrospective fashion.

Patient characteristic data is collected along with hospitalization and posthospitalization data. Patient characteristic data collected includes age, comorbidities, previous intra-abdominal surgery, weight, height, body mass index (BMI), medical insurance status, ASA score, and performance status. Hospitalization details included the procedure performed, type of incision (transverse or midline), operating time, complexity of surgery (simple versus complex), operation category (elective versus emergency), wound infection risk (clean versus clean contaminated versus contaminated), intraoperative estimated blood loss (EBL), whether a transfusion was required, the preoperative haemoglobin (Hb), postoperative Hb and the Hb change, whether patients tolerated early oral feeding (EOF) and if the patient received cyclooxygenase inhibitors (COX inhibitors). All inpatient complications were collected, including modified Royal Australian and New Zealand College of Obstetricians and Gynecologists (RANZCOG) Quality Indicators. Date of admission and date of discharge were used to calculate LOS. Posthospitalization admissions and complications were also recorded. Not all data fields were collected from initiation of the audit. In year 3 “Suitability for D/C on day 2” was included. From year 4 the following data fields were added to the audit: “ASA score,” “comorbidities,” “previous intra-abdominal surgery,” “operation category,” “wound infection risk”.

## 4. Definitions

Simple surgery was defined as simple type 1 hysterectomy or adnexal surgery where formal retroperitoneal dissection or ureteric dissection was not performed. All surgeries where at least a formal pelvic sidewall dissection was undertaken, including bowel, bladder, nodal dissection, and omentectomy were classified as “complex.” Transverse incisions were classified according to the incision in the skin, irrespective of whether it was of Maylard or Pfannenstiel type.

Patients were classified on final pathological determination as either “benign” or “malignant”. Patients with proliferating or borderline ovarian tumours were classified as “benign” as were patients with complex atypical endometrial hyperplasia and patients with cervical dysplasia needing definitive treatment and patients undergoing definitive hysterectomy for persistent gestational trophoblastic disease. Patients with malignant pathology were routinely reviewed 2 weeks postoperatively and then regularly thereafter; whilst those patients with benign pathology were reviewed 2–4 weeks after operation.

## 5. Statistical Analysis

Ethics approval was granted to allow review and presentation of the data as a clinical audit. Statistical analysis including descriptive statistics, *t*-test, and ANOVA for nominal variables and Chi-squared test for categorical data.

## 6. Elements of a Fast Track Surgery Program

### 6.1. Preoperative Patient Assessment and Counseling

Crucial to the process and success of FTS is the preoperative counseling and assessment of patient fitness for surgery. Medical risk assessment and optimization of medical care and appropriate preoperative medical consultations including a preoperative anesthetic assessment will allow the patient to enter hospital and surgery in an optimized condition, resulting in an overall reduction in preoperative morbidity [27].

During the assessment and counseling period, medical and nursing staff counsel and educate the patient as to what they are to expect whilst in hospital and what is expected of them as far as early mobilization, early oral feeding, pain control and expected date of discharge and discharge planning, and the rationale for such an approach. If patients “expect” to remain in hospital for 5–7 days, then irrespective of their convalescence, they will remain in hospital for that period of time. Callesen et al. found that by merely recommending a short convalescence, the time to resume work after hernia repair could be dramatically reduced [28]. Before their surgery, patients are comprehensively prepared with a package of educational material, both written and verbal about their “surgical journey” and an objective assessment of their fitness to undergo the operation they need. Preoperative patient counseling and patient education have also been shown to have an effect on perioperative analgesia requirements [8, 29].

During this preoperative assessment period, preoperative discharge planning is commenced. The process is important as it allows hospital staff and family and carers to make the necessary arrangements as to the expected date of discharge, what are the criteria for discharge and that a large proportion of patients will meet these criteria earlier than expected and that arrangements will need to be made for whenever the patient is discharged from hospital.

### 6.2. No Premedication

Traditionally sedative premedication has been prescribed to decrease patient anxiety. Such practice is rarely if ever employed in FTS. By avoiding long acting preoperative sedative premedication, the outcomes of the FTS program are enhanced by allowing early patient mobilization, initiation of early oral feeding and catheter removal [8].

### 6.3. Preemptive Analgesia

Preemptive analgesia is well documented in cancer pain management and is becoming increasingly important in the management of perioperative acute pain. By initiating analgesia (and anaesthesia) prior to the initiation of noxious stimuli, peripheral, and central nervous system pain receptor activation is blocked, reducing the production of and activity of pain neurotransmitters and processing can be modified resulting in improved short-term and long-term pain control and reduced side effects from narcotic analgesics.

For preemptive local anaesthesia, commonly our TAP blocks are performed just after intubation and just before surgery is commenced. For preemptive analgesia, we use Gabapentin 900 mg oral 1 hour prior to surgery. A large number of randomized controlled trials have confirmed preoperative gabapentin results in decreased analgesic requirements [30–35]. Others have used ibuprofen and ketorolac and clonidine. Preemptive gabapentin appears not to be effective however in reducing postoperative pain [36]. 

Further preemptive analgesia is initiated with COX-2 inhibitors given intravenously pre- and intraoperative (parecoxib) with intravenous paracetamol [37]. In addition intravenous (and oral) paracetamol has opioid sparing effects [38]. Oral meloxicam is continued daily in the postoperative period whilst in hospital. The tolerability and effectiveness have been well documented [39, 40].

### 6.4. Minimising Fasting

Traditionally patients are fasted to reduce the risk of aspiration. Based upon current guidelines adults should fast at least 2 hours from clear liquid intake [41]. The evidence for the use of perioperative carbohydrate rich oral supplements is encouraging but lacking in quality [42].

To reduce the risk of postoperative ileus, we use a perioperative gastrointestinal strategy of minimizing fasting, avoiding NG tubes, and avoiding mechanical bowel preparation. The mechanisms of postileus are complex and involves an interplay of gastrointestinal inflammatory response, an irritation of peritoneal surfaces stimulating afferent reflexes and the interplay of pharmacological agents, in particular narcotics resulting in an uncoordinated peristaltic response probably as a consequence of activation of gastrointestinal mu-opioid receptors which inhibit the release of acetylcholine from the mesenteric plexus [43]. Myoelectric studies of contractility of the stomach, small and large bowel has shown that the stomach and small bowel contractility returns to normal soon after surgery, the colon may take a number of days to do so. Anaesthesia alone has little effect on intestinal peristalsis but when combined with surgery bowel motility is significantly impaired. In addition, it would appear that the length of surgery and the amount of intestinal manipulation have little impact on peristalsis; however, retroperitoneal dissection has a significant effect on developing colonic stasis [44, 45].

### 6.5. Avoidance of Mechanical Bowel Preparation

With traditional surgical care models, mechanical and antibiotic bowel preparation was considered the norm for patients undergoing surgery for suspected or confirmed gynaecological malignancy particularly if bowel resection was being contemplated. The rationale was to reduce the risk of anastomotic leakage and perioperative infectious morbidity by reducing faecal flora [46]. A recent Cochrane review of 13 prospective studies of patients undergoing large bowel resections showed no difference in anastomotic leakage rates between patients having bowel preparation (4%) compared to those not having bowel preparation (3%) [47]. The authors have indicated a “selective” use of mechanical bowel preparation, and our protocol is to consider mechanical bowel preparation where a low rectal anastomosis might be required, where there is significant tumour burden, with ascites and low serum albumin, all potentially in their own right of increasing leak rate and infectious morbidity.

### 6.6. Venous Thrombo-Embolism (VTE) Prophylaxis

It is well established that patients with cancer have an increased risk of VTE development. Confounding and contributing factors include increasing age, duration of hospital stay, mobility of the patient after surgery, duration and type of surgery performed, stage of disease, residual disease after surgery, patient performance status, previous history of VTE, and postsurgery treatment with hormone therapy, anti-angiogenic, cytokine therapies, and chemotherapy. Generally recurrence and survival is reduced in cancer patients who develop VTE.

The impact of surgery on thrombosis risk depends upon the site of malignancy and type of surgery. The risk is highest for those cancer patients undergoing major abdominal or pelvic surgery. Cancer patients undergoing gynaecological surgery are also at a high-risk or VTE development but this risk has been difficult to quantify. There is a high incidence of late thrombosis in surgical cancer patients, with up to 40 percent of VTE events occurring more than 21 days after surgery [48].

However it needs to be reemphasized that not all cancers and not all surgeries have the same thrombogenic potential. Indeed, not all patients, with the same cancer, having the same surgery have the same thrombogenic potential. That overall the incidence of symptomatic VTE in the gynaecological oncology surgical patient is low and that none of the reported data relates to patients enrolled in FTS programs where early mobilization is a key and central component.

Most studies reporting incidence of VTE are compromised by design flaws and low numbers of patients with gynaecological cancers and confounding variables. VTE is usually initiated prior to surgery and that whilst a large proportion are diagnosed in the immediate period after discharge from hospital, a significant proportion occur late with 40% being diagnosed after 21 days in the RISTOS project [48].

Overall the incidence of clinical VTE in gynaecological oncology is low and is in the order or 1–3%. In the Million Women Study the incidence rate per 1000 person months after gynaecological surgery was 0.99, with 1 in 365 (0.3%) developing a VTE over 12 weeks [49]. The incidence of VTE was 1.2% after hysterectomy for malignancy with 0.7% occurring after discharge in a large California Patient Discharge Data Set [50], whilst 2% of gynaecological patients in the @RISTOS study developed VTE [48]. In a single institution study of patients with gynaecological cancers 3.1% developed VTE in the Mayo Clinic Study, but this result needs to be tempered by the fact that it included patients having post surgical chemotherapy for ovarian cancer [51].

All patients in our program are commenced on perioperative enoxaparin sodium 20–40 mg SCI which is continued until discharge. The manufacturers recommended the first dose be given 12 hours preoperatively. As this is usually impractical, we usually administer the first dose the night of surgery. Whilst not routine, selected high risk patients are offered extended enoxaparin sodium prophylaxis. Intraoperatively mechanical sequential compression devices are utilized and currently these remain deployed throughout the duration of hospitalization except during times of mobilization. All patients have knee high TED stockings fitted and worn postoperatively for at least 1 month [52]. These recommendations are not too dissimilar to the Australian National Health and Medical Research Council (NHMRC) Guidelines. In the NHMRC Guidelines, it is recommended (level B grade of recommendation) that patients undergoing gynaecological surgery, including complex curative surgery for cancer to commence pharmacological thrombo-prophylaxis and to continue this for up to one week or until the patient is fully mobile [53].

Antithrombotic prophylaxis however remains an area of concern for us. We recognize that our incidence of VTE is low, and this may be in part due to the early mobilization of the FTS program. However due to reports of VTE occurring post discharge [48, 54, 55] in selected high risk patients, extended enoxaparin sodium prophylaxis is employed. Such high risk factors include a previous history of VTE, morbid obesity, prolonged hospitalization and the presence of bulky residual disease at the completion of surgery [51, 56]. Fearon and colleagues have recommended patients should receive VTE prophylaxis according to the local peer-review protocol [57]. Whether a full 7–10 days of chemical prophylaxis is required or just until fully mobile in a “fast-track” population is unclear. It is reassuring that in breast cancer patients treated on a clinical pathway, venous thromboembolism (VTE) is rare when they had mechanical antiembolism devices and early ambulation in the postoperative period [58].

### 6.7. Intraoperative Analgesia and Regional Anaesthesia

Pain postoperatively has a negative impact on patient quality of life, their ability to mobilize, commence early oral feeding, have their urinary catheter removed and successfully use incentive spirometry, all resulting in an increased catabolic stress response and increased length of stay [59].

The use of regional anaesthetic techniques after fast track hysterectomy has resulted in substantial advantages, providing faster recovery and shorter sick leave [60].

Transverse abdominal plane blocks have been utilized extensively and found to be safe and effective but our understanding of them is limited and further studies need to be performed [61–63]. TAP blocks are safe, reduce post-operative morphine requirements, nausea and vomiting and possibly the severity of pain after abdominal surgery. It should be considered as part of a multimodal approach to anaesthesia and enhanced recovery in patients undergoing abdominal surgery.

Regional anaesthesia techniques are important in the overall scheme of pain reduction, as they results in decreased opioid requirement [27, 64–67].

### 6.8. Avoidance of Nasogastric (NG) Tubes

In parallel with the tradition of avoiding early oral intake, the use of nasogastric tubes was often considered routine after major abdominal surgery. The rationale was to assure decompression of the stomach to reduce the risk of aspiration, nausea and vomiting and ileus postoperatively as well as wound dehiscence. The routine use of NG tubes has been shown to have little impact on emesis and often results in an increased risk of aspiration pneumonia [68] and has little impact in the rates of dehiscence [69]. In the SGOG FTS protocol routine insertion nasogastric tubes is never undertaken. Rarely a distended stomach noted intraoperatively will be decompressed during the surgical procedure but the NG tube is removed at the completion of surgery and prior to extubation. Nasogastric tubes are only rarely inserted postoperatively for patient with abdominal distention and vomiting. Abdominal distention without vomiting is not an indication for NG tube insertion.

### 6.9. Short Acting Anaesthetics

The goal of anesthesia is to provide and maintain a safe environment of stable unconsciousness for surgery whilst maintaining as much as possible normal physiology. Extrapolation of techniques employed for ambulatory surgery to complicated surgical procedures and are paramount in the safe implementation of FTS. Techniques include the introduction of short acting volatile anaesthetics, short acting opioids, and analgesics and muscle relaxants [70].

### 6.10. Avoid Fluid Overload

Over the past few decades, the philosophy of perioperative fluid management has fluctuated between overhydration to the philosophy of under hydration/fluid restriction or “running dry”. Length of stay has not been reduced by the latter philosophy [71]. Current fast track principles are to maintain normal or euvolaemia throughout the entire perioperative period. Goal directed fluid therapy to maintain stroke [71] volume should be individualized [72, 73].

### 6.11. Early Catheter Removal

Urinary catheters are inserted prior to surgery for essentially two reasons. Firstly to monitor urine output as a surrogate of patient hydration and secondly as a “kindness gesture” to avoid the immediate postoperative patient from having to use a bed pain or toilet. However with the advantages of a FTS, allowing adequate pain relief and early mobilization, early urinary catheter removal has been found to be associated with a reduced length of stay without and increase in complications [74]. A novel approach is the immediate discontinuation of the urinary catheter at the completion of surgery [75].

### 6.12. Avoid Drains

Utilizing meticulous dissection and haemostasis has allowed a review of the almost routine use of pelvic drain policy that has occurred over the last decade. The routine use of peritoneal drains with abdominal or pelvic surgery has not been shown to have a positive effect on morbidity or anastomotic leakage rates. Our policy is only to use drains when the likelihood of a pelvic collection is increased and where haemostasis is suboptimal despite attempts at meticulous haemostasis [76, 77].

### 6.13. Temperature Regulation and Intraoperative Warming

Changes in body temperature during surgery particularly during prolonged surgery and in the elderly, frail and thin results in an increased risk of development of complications including wound infection, cardiac events, coagulopathy and impaired oxygen transportation, and postanaesthetic recovery [78, 79]. Routine use of rewarmers such as Bair Hugger is an integral part of maintaining patient temperature during surgeries and thus minimizing the effects of prolonged hypothermia.

### 6.14. Post-Op Care pathways

Care pathways (CP) allow a standardization of individual facets of postoperative care and have been shown to improve many aspects of the perioperative process including length of stay and tolerability of surgery [80, 81].

### 6.15. Nonopioid Analgesia and NSAIDs

Postoperative pain control is initiated in the intraoperative setting and continues throughout the postoperative period and after discharge. Adequate pain control ensures early mobilization, reduction in morbidity such as atelectasis and VTE, reduced hospital stay, reduced hospital costs and increased patient satisfaction [82–84]. A multidisciplinary approach combining the expertise of the anaesthetist and the acute pain service is useful and important in this regard.

### 6.16. Early Oral Feeding

Paramount to the success of a FTS or ERAS program is the implementation of “early oral feeding” (EOF). Traditionally patients undergoing major abdominal surgery had been kept “Nil By Mouth” (NBM) for a variable period of time after surgery. Usually once bowel sounds were auscultated and/or flatus passed, then oral intake was commenced. Again traditionally this had been in a graduated fashion with sips of water, clear liquids, free fluids, light diet, and then finally regular diet.

Concern is often raised that introduction of full diet before the return of bowel function will result in a significant increase in abdominal distension, ileus, bowel obstruction, and prolonged length of stay. Data supporting this long held practice is scarce. There is increasing evidence to the contrary, that the early introduction of enteral nutrition reduces morbidity without increasing ileus or obstruction rates. The introduction of a low residue diet 6 hours after major gynaecological surgery has not been shown to be associated with increased gastrointestinal complaints including ileus [85], indeed it has been shown to aid in a decreased LOS [86]. Early enforced mobilization and enteral nutrition in patients having colonic resection not only reduces postoperative ileus but also cardiopulmonary complications [87].

EOF is well accepted by patients and is associated with a reduction in abdominal distention, nausea, vomiting, and postoperative ileus [88, 89].

In addition a number of other initiatives can be employed to reduce the likelihood of development of postoperative ileus. For instance gum chewing of sugarless gum for 15–30 minutes at least 3 times daily has been demonstrated safe, effective with a positive effect at decreasing postoperative ileus [90]. The purported mechanism is believed to be the activation of cephalic-vagal pathway that stimulates intestinal myoelectric activity of the gastrointestinal mu-opioid receptors.

### 6.17. Postoperative Nausea and Vomiting Protocol (PONV Protocol)

Despite advances in anaesthetic techniques, preemptive medication, advances in surgical techniques, postoperative nausea and vomiting (PONV) remains a common problem occurring in about a third of all surgeries. Amongst high-risk patients as many as 70% of patients will experience PONV. Postoperative nausea and vomiting can delay hospital discharge or result in unplanned admission. Vomiting can stress wounds, imbalance body electrolytes, and cause bleeding.

The cause is multifactorial with patient factors important along with medication and surgical factors. Females, non-smokers and patients with a history of motion sickness are predisposing patient related factors increasing the risk of PONV. Anaesthetic factors include the use of volatile anaesthetic agents, nitrous oxide, and perioperative opioid use whilst surgical factors include the duration and type of surgery undertaken for example laparoscopy and strabismus and middle ear surgery [91].

When single agent therapy is ineffective, combination therapy has been shown to be effective. The weight of evidence clearly shows that if an agent is ineffective against PONV, repeating a second dose of that agent is unlikely to increase efficacy. In fact, in addition to increasing cost, it is likely to increase the incidence of side effects. Therefore, the common practice of giving a second dose of the serotonin antagonists is not only expensive, but also useless. The key to choosing additional agents when a single agent fails is to choose an agent with a different mechanism of action. Nausea and vomiting involve multiple receptors, neurotransmitters, neural pathways, and so forth. Choosing agents that affect different receptors is the key to successful combination therapy. For example, if the serotonin receptors have already been blocked, consider adding an anticholinergic, anti-dopaminergic, or antihistamine.

The PONV Treatment Protocol at our institution is a stepwise protocol with prochlorperazine, tropisetron, and droperidol and can by initiated by the ward nurse without countersigning.

### 6.18. Incentive Spirometry

The perioperative use of incentive spirometry (along with early mobilization and head of bed elevation) has been shown to have a positive effect on preventing atelectasis and pneumonia [92]. Our protocol recommendation is for the device to be used 6 times per hour.

### 6.19. Early Mobilization

Along with early oral feeding, early mobilization is one of the central components for a successful FTS program. Mobilization will counter effects of bed rest on muscle loss and weakness, will allow expansion of lung bases and the effect on impaired pulmonary function, tissue oxygenation and increased insulin resistance as well as allow increased blood circulation reducing the risk of VTE [92, 93].

### 6.20. Laxatives

The concept of fast-track multimodal rehabilitation appears to be beneficial in patients operated for ovarian malignancy, as hospital stay and medical morbidity are reduced [94]. Although the use of laxatives has been incorporated into several fast-track abdominal and gynecologic programs with reported success, until recently no information was available from double-blind, placebo-controlled randomized trials. In a recently published RCT, it was found that early postoperative laxative use significantly improved recovery of gastrointestinal function and reduced median time to first defecation with 24 hours without an adverse effect on pain scores or postoperative nausea and vomiting [95]. In patients undergoing radical hysterectomy for cervical cancer, aggressive bowel stimulation with Fleet Phospho-Soda and early feeding resulted in early return of bowel function and early discharge without significant intestinal complication [96].

### 6.21. Nurse Led Follow-Up Clinic

The goal of fast track programs is to provide an enhanced recovery after surgery, early discharge with reduced complications, reduced readmission rate and enhanced quality of life and satisfaction [97]. To ensure these objectives are met, our patients are contacted by our Clinical Nurse Consultant via telephone after discharge and are reviewed 1 week post-discharge in a purpose designed “Nurse Led Follow Up Clinic”. Patient satisfaction appears to be enhanced with improved quality of life and nurse satisfaction is also enhanced [60, 98].

### 6.22. Audit

The process of clinical audit is fundamental to clinical governance, the process by which clinicians improve the quality of the care they provide. Clinical audits are powerful tools as they present data on all patients who underwent surgery/laparotomy with no exclusions and as such represents “real life” experience. The development of our program and audit of our experience is summarized with comment on the applicability of FTS for general gynaecology. This rolling audit encompasses almost 5 years of experience with a FTS program and as such is one of the largest in the literature.

## 7. Rationale and Initial Experience

In 2008, an FTS program was initiated at the Sydney Gynaecological Oncology Group (SGOG). At the completion of that year the outcomes of those patients managed by FTS were compared to patients, not managed by FTS. Our initial experience showed that those patients managed by FTS were able to be discharged with a reduced LOS, without an increased readmission rate and with comparative outcomes to non-fast tracked patients. Based upon this preliminary data, the concept was adopted as standard of care [14].

## 8. Overview and Review of All Data

Fast-track surgery programs have been widely reported, but their incorporation into mainstream surgery and gynaecology in Australia has been slow. Whilst the concept has not been tested in an RCT fashion, extensive data would imply a benefit for the patient with reduced morbidity and a benefit for the health care provider and institution with early discharge and resultant cost saving.

The process of clinical audit is fundamental to clinical governance, the process by which clinicians improve the quality of the care they provide. Clinical audits are powerful tools as they present data on all patients who underwent surgery/laparotomy with no exclusions and as such represents “real life” experience. The development of our program and audit of our experience is summarized with comment on the applicability of FTS for general gynaecology.

Since the introduction of the program in 2008, 389 have been entered on a rolling clinical audit of FTS for laparotomy. There were no exclusions. Median age was 55 years (range: 20.1–87) with 227 (58%) having confirmed malignancy. Thirty-eight percent of patients had prior abdominal surgery and the most common site of pathology was ovarian (51%), uterine corpus (39%), and uterine cervix (9%). Overall 162 (42%) had benign pathology and of those with malignant disease, 34% had stage I disease, 4% had stage II disease, 17% had stage III disease, and 4% had stage IV disease.

Surgery was deemed to be complex in 348 (89%), with lymph node sampling or dissection performed in 68 (17%). Vertical midline incisions accounted for 90% of the incisions.

Two hundred and sixty-two (67%) patients were considered to be fully active and assigned a “0” performance status, whilst 95 (24%) had light restrictions and assigned a performance status of “1”. Twenty nine (7%) were assigned a performance status of 2, not being able to work and being ambulatory 50% of the time whilst 3 patients had limited self care and were assigned a performance status of “3”. Forty one percent of patients had an ASA score of 1, 36% had an ASA of 2 and 23% had an ASA of 3. One patient had an ASA of 4.

Median body mass index (BMI) was 26.5 (range: 16.9–68.8) with 114 (29%) considered overweight and 121 (31%) classified as obese.

The volume of surgery over the period of the rolling audit was 73 surgeries performed in year 1, 99 surgeries performed in year 2, 79 surgeries performed in year 3, 96 surgeries performed in year 4 and for 6 months of year 5, 42 surgeries were performed. Surgery was considered complex in 348 (89%) patients and 97% were considered elective.

Median operating time was 2.25 hours (range: 0.75–10). One hundred and sixty one patients (41%) had operations that lasted 2 hours or less, 173 (44%) had surgery durations between 2-3 hours and 45 (12%) had surgeries lasting 3-4 hours. Ten patients (2.6%) had surgeries lasting longer than 4 hours. Nineteen (5%) patients had a formal radical hysterectomy or trachelectomy performed for either an early cervical cancer or stage II corpus cancer.

Median estimated blood loss (EBL) at surgery was 175 mL (range: 10–3500) and median Hb change (preop to postop) was 11 g/L.

Median LOS was 3 days (range: 2–27) with average LOS of 3.5 days. Overall 110 (28%) patients were discharged on day 2. A further 21 patients were suitable to be discharged on day 2 but for social reasons were unable to be discharged. Twenty (5%) patients had a LOS of greater than 7 days. Sixteen (4%) patients were readmitted and the reasons outlined in Table 1. Whilst a number of variables were associated with reduced LOS on univariate analysis, on multivariate analysis, benign pathology, shorter operating time and the ability to tolerate early oral feeding were found to be significant (Table 2).

### 8.1. Comment

In support of our previous work and the work of others, the data overwhelmingly shows the tolerability and success of a FTS program in patients referred with suspected or confirmed gynaecological malignancy. Despite complex surgeries being performed in many patients with significant comorbidities, the median LOS was 3 days with 28% being discharged after major surgery on day 2. The readmission rate of 4% is considered acceptable with the majority of the reasons for readmission not related to the FTS program (Table 1). Readmission rates are an important outcome measure to document the success of a FTS program. At the patient level, reported independent risk factors for readmission include older age, male sex, black race, lower socioeconomic status, urgent or emergency surgery, complex comorbidities, perioperative complications, open (versus laparoscopic) surgery, and increased length of stay for the index hospitalization [97]. Evaluating only those fast track elements that were successfully achieved, early oral feeding, early mobilization, laparoscopic surgery, and female sex were independent determinants of enhanced recovery [99].

## 9. Patients with Benign Gynaecological ****Pathology

With the huge volume of benign gynaecological surgery performed throughout the world, the impact of FTS programs will be far greater in benign gynaecology than in gynaecological oncology. Despite the overall favorable results reported below, the fact needs to be reemphasized is that patients operated upon by the author represent, a high risk group of patients with suspected malignancy and as such are of higher risk, often older and often with more comorbidities.

Overall 162 (42%) patients had benign pathology. Median age was 49 years (range: 20.1–80.1) with 76 (47%) aged 50 or older and 3 (1.8%) aged over 75 years. Thirty eight percent of patients had prior abdominal surgery and the most common site of pathology was ovarian (67%), uterine corpus (25%) and uterine cervix (7%). Surgery was deemed to be complex in 126 (78%). Vertical midline incisions accounted for 82% of the incisions and 97% of operations were considered elective.

One hundred and thirty two (81%) patients were considered to be fully active and assigned a “0” performance status, whilst 19 (12%) had light restrictions and assigned a performance status of “1”. Ten (6%) were assigned a performance status of 2, not being able to work and being ambulatory 50% of the time whilst 1 patient had limited self care and were assigned a performance status of “3.” Fifty nine percent patients had an ASA score of 1, 29% had an ASA of 2 and 12% had an ASA of 3.

Median body mass index (BMI) was 26.4 (range: 18.1–68.8) with 47 (29%) considered overweight and 53 (33%) classified as obese.

Median operating time was 2 hours (range: 0.92–5.5). Three (2%) patients had operations lasting less than 1 hour, 92 patients (57%) had operations that lasted between 1-2 hours, 55 (34%) had surgery durations between 2-3 hours and 8 (5%) had surgeries lasting 3-4 hours. Four patients (2.5%) had surgeries lasting longer than 4 hours. Median EBL at surgery was 150 mL (range: 10–3500) and median Hb change (preop to postop) was 12 g/L.

Median LOS was 3 days (range: 2–20) with average LOS of 3.2 days. Overall 68 (42%) patients were discharged on or before day 2. A further 5 patients were suitable to be discharged on day 2 but for social reasons were unable to be discharged. Five (3%) patients had a LOS of greater than 7 days.

Three (1.9%) patients developed wound infections, 1 bladder injury and 4 (2.5%) patients readmitted. One patient (0.6%) experienced a VTE, 4 (2.5%) received blood transfusions, one transfused preoperatively (Table 3).

### 9.1. Comment

As outlined in the introduction to this section despite the patients being described as benign, this did not correlate with being of low risk. One-third of all benign patients were obese and 19% had a nonzero performance status and 41% had and ASA score greater than 1. This is demonstrated by the fact that median operating time was 2 hours and 4 patients had surgeries lasting longer than 4 hours. Despite this, median LOS was 3 days and a staggering 42% were discharged on day 2. The complication rate was very low and only 2.5% patients were readmitted. One can only assume that in a “routine” general gynaecology practice with much lower risk patients the LOS and outcomes would be even better.

## 10. Uterine Corpus Cancer

Endometrial cancer is the most common gynaecological cancer affecting women, and with an increasing incidence, a safe, cost effective, and tolerated management is important [100]. The treatment remains removal of the uterus and adnexa, and this can be accomplished via laparotomy, vaginally, totally laparoscopic, laparoscopically assisted or robotically. Surgical staging to define the extent of disease may be added to hysterectomy however the rationale for this and data on survival impact is often debated [101]. The following series represents almost 5 years of continuously acquired data on the surgical management of uterine corpus cancer via laparotomy and selective surgical staging.

Median age was 60.5 years (range: 35.1–87) with 92 (82%) older than 50 years and 13 (12%) older than age 75 years. Thirty four percent of patients had prior abdominal surgery, 74% had stage I disease, 11% had stage II disease, 10% had stage III disease, and 5% had stage IV disease. Lymph node sampling or dissection was performed in 43 (38%). Vertical midline incisions accounted for 99% of the incisions.

Fifty six (50%) patients were considered to be fully active and assigned a “0” performance status, whilst 42 (38%) had light restrictions and assigned a performance status of “1”. Thirteen (12%) were assigned a performance status of 2, not being able to work and being ambulatory 50% of the time whilst 1 patient had limited self care and were assigned a performance status of “3”. Twenty two percent of patients had an ASA score of 1, 44% had an ASA of 2 and 31% had an ASA of 3. One patient had an ASA of 4. Median body mass index (BMI) was 28.8 (range: 17.5–52.2) with 37 (33%) considered overweight and 49 (44%) classified as obese.

Median operating time was 2.33 hours (range: 0.75–5). Thirty seven patients (33%) had operations that lasted 2 hours or less, 65 (58%) had surgery durations between 2-3 hours and 9 (8%) had surgeries lasting 3-4 hours. One patient had surgery lasting longer than 4 hours having an exenterative type procedure for primary serous carcinoma with a background of abdominoperineal resection and pelvic irradiation for colon cancer previously. Median EBL at surgery was 150 mL (range: 10–900) and median Hb change (preop to postop) was 9.5 g/L.

Median LOS was 3 days (range: 2–16) with average LOS of 3.4 days. Overall 20 (18%) patients were discharged on day 2. A further 11 patients were suitable to be discharged on day 2 but for social reasons were unable to be discharged. Four (3.6%) patients had a LOS of greater than 7 days.

There were no intraoperative blood transfusions and 1 preop and 1 postop transfusion, none receiving more than 2 units of packed cells. All patients tolerated EOF, there were 5 (4.5%) wound infections, 1 febrile morbidity, and 2 (1.8%) cases of VTE, 1 patient suffered a bladder injury, 2 (1.8%) patients returned to the operating room and 3 (2.7%) admitted for monitoring in ICU (Table 3).

There were in total 7 (6.3%) readmissions. One patient with a wound infection, readmitted and returned to the operating room for debridement. A second obese patient with wound infection on extended Clexane prophylaxis. The third patient was electively readmitted for trial of void, another was readmitted and returned to the operating room for resuture of vaginal vault. One patient was readmitted with constipation and another with viral gastroenteritis 2 weeks after discharge. 

One patient accounted for a total of 5 complications. She was a 61-year-old, obese Jehovah's Witness described above with a past history of abdomino-perineal resection and pelvic irradiation for colon cancer. She was diagnosed with a serous corpus cancer and her uterus was fixed onto the sacrum as a consequence of previous treatments. Intraoperatively ureteric stents were inserted. Post operative bleeding from ureteric stents required her return to the operating room by the Urology team for removal of ureteric stents, and monitoring in ICU and subsequent readmission to hospital for management of an electrolyte disturbance. 

A second patient accounted for 3 complications. She was a 60-year-old morbidly obese woman who underwent repair of an incisional hernia by general surgical colleagues at the same time as her uterine cancer surgery with extensive soft tissue mobilization and was readmitted 3 weeks after surgery with a wound infection that required debridement in the operating room and VAC dressing placement.

### 10.1. Comment

This study provides the first real base-line or “recognized standard” on laparotomy patients managed by FTS for the surgical management of uterine cancer. During the study period there were no patients who underwent laparoscopy and thus there is no selection bias involved. As such this audit represents extensive experience of fast track surgical care in patients with uterine malignancy managed by laparotomy and thus serves as a “recognized standard.”

The extended experience confirms our earlier work that the majority of patients can complete an FTS program, with minimal morbidity and a low incidence of readmission and as a consequence, a shorter hospital stay [14]. Patients with corpus cancer are more likely to be obese with a poor performance status or ASA score and to have had prior abdominal surgery. Almost 40% underwent regional lymph node assessment adding complexity to their surgery. Despite this operating times and EBL were quite acceptable and the median LOS was also acceptable at 3 days. The complication rate from surgery and readmission rate was higher than expected. However data from clinical audits can bias or skew reporting as all patient data is collected. In this series one patient in particular accounted for 5 complications and her case was quite unusual and complex. Otherwise FTS in patients with corpus cancer remains safe with good outcomes reported [11, 15].

## 11. Ovarian Cancer

Epithelial ovarian cancer accounts for 80% of all ovarian cancers, is more common in industrialized countries affecting middle and upper classes more commonly. There are just over 1200 cases diagnosed yearly and 850 deaths. Indeed ovarian cancer continues to hold the dubious distinction as the most deadly of all the gynaecological cancers with more women dyeing from ovarian cancer than from cervical and uterine cancers combined. Whilst ovarian cancer is primarily a disease of older women, it is salient to remember that 1 in 5 will be under 50 and 1 in 10 under the age of 40 at diagnosis. Standard management is surgical staging in apparent early stage disease and adjuvant therapy for high risk early stage patients. Exploratory laparotomy and surgical cytoreduction or debulking is the mainstay of treatment in advanced stage disease.

In this audit eighty nine patients were diagnosed with malignant ovarian tumours. Median age was 57.5 years (range: 22.1–85) with 64 (72%) over 50 and 7 (8%) over age 75. 52 percent of patients had prior abdominal surgery, and 30% had stage I disease, 3% had stage II disease, 57% had stage III disease and 9% had stage IV disease. Lymph node sampling or dissection performed in 8 (9%). Vertical midline incisions accounted for 98% of the incisions.

Fifty one (57%) patients were considered to be fully active and assigned a “0” performance status, whilst 31 (35%) had light restrictions and assigned a performance status of “1.” 6 (7%) were assigned a performance status of 2, not being able to work and being ambulatory 50% of the time whilst 1 patient had limited self care and were assigned a performance status of “3.” Eighteen percent of patients had an ASA score of 1, 41% had an ASA of 2 and 41% had an ASA of 3. Median body mass index (BMI) was 24.6 (range: 16.9–62) with 27 (30%) considered overweight and 15 (17%) classified as obese.

Median operating time was 2.5 hours (range: 1–5.5). Twenty three patients (26%) had operations that lasted 2 hours or less, 46 (52%) had surgery durations between 2-3 hours and 18 (20%) had surgeries lasting 3-4 hours. Two patients (2%) had surgeries lasting longer than 4 hours. Median EBL at surgery was 250 mL (range: 25–3500) and median Hb change (preop to postop) was 11 g/L.

Median LOS was 4 days (range: 2–14) with average LOS of 4.2 days. Overall 14 (16%) patients were discharged on day 2. A further 3 patients were suitable to be discharged on day 2 but for social reasons were unable to be discharged. 9 (10%) patients had a LOS of greater than 7 days.

Seven (8%) patients required blood transfusions, 2 of whom required greater than 2 units of packed cells. 85% tolerated EOF, there was 1 (1%) wound infection, 1 (1%) febrile morbidity, no urinary bladder, ureter, bowel or vascular injury, 5 (6%) readmissions, 1 (1%) VTE. There were no returns to the operating room, and 1 patient was admitted to ICU postop (Table 3).

### 11.1. Comment

As would be expected for an audit on ovarian cancer, most patients had advanced stage disease and almost all patients had complex surgeries performed. Most patients had a zero performance status with only 17% considered obese. Median operating time was not excessive, nor was EBL at surgery but LOS was surprisingly longer than the norm, at 4 days. The rate of blood transfusion in this subgroup was higher (8%), as was the readmission rate (6%).

## 12. Cervical Cancer

Whilst one of the most common cancers affecting women worldwide, in developed countries with national screening programs, the incidence of cervical cancer has dramatically declined [102].

During the audit period, 21 patients underwent laparotomy for an invasive cervical cancer. Median age was 41 years (range: 25–71.2) with 6 (29%) over age 50. Fourteen percent of patients had prior abdominal surgery. Twenty (95%) had stage I disease and 1 (4.5%) patient with stage III disease underwent an exploratory laparotomy. Lymph node sampling or dissection performed in 14 (67%). Vertical midline incisions accounted for 67% of the incisions. All 21 patients were considered to be fully active and assigned a “0” performance status. Eighty six percent patients had an ASA score of 1 and 14% had an ASA of 2. Median body mass index (BMI) was 21.8 (range: 16.9–38.1) with 3 (14%) considered overweight and 3 (14%) classified as obese.

Median operating time was 3 hours (range: 1.5–4.6). Five patients (24%) had operations that lasted 2 hours or less, 6 (29%) had surgery durations between 2-3 hours and 8 (38%) had surgeries lasting 3-4 hours. Two patients (10%) had surgeries lasting longer than 4 hours.

Fourteen (67%) patients had a formal radical hysterectomy or trachelectomy performed for either an early cervical cancer. Median EBL at surgery was 150 mL (range: 50–650) and median Hb change (preop to postop) was 10 g/L.

Median LOS was 3 days (range: 2–10) with average LOS of 3.3 days. Overall 6 (29%) patients were discharged on day 2. A further 1 patient was suitable to be discharged on day 2 but for social reasons were unable to be discharged. One (5%) patient had a LOS of greater than 7 days.

All tolerated EOF, there were no transfusions, no readmissions and no return to operating room. The only significant complication being a transient obturator nerve injury, with all symptoms and signs settling prior to discharge (Table 3).

### 12.1. Comment

With the advent of successful screening programs, the requirement for exploratory surgery for an invasive cervical cancer has dramatically declined. Of all the subgroups analyzed, cervical cancer had the fewest patients. Cervical cancer patients tend to be younger, thinner, fitter and have earlier stage disease. The majority underwent formal surgical lymph node assessment. Surgical complications, duration of surgery and LOS were well within expectations with median LOS of 3 days, no transfusions, no readmissions and no persisting postop complications.

## 13. Radical Hysterectomy for Cervical Cancer

The surgical management of cervical cancer may extend from a simple of Type 1 hysterectomy without node dissection for an early stage IA1 cancer, through to a formal Type 3 radical hysterectomy with pelvic and low paraaortic lymph node dissection for stage IB1 disease. In addition young women with a desire to retain their fertility potential may also undergo fertility sparing radical trachelectomy with lymph node dissection [103].

During the audit period, 14 patients with stage I cervical cancer underwent either radical hysterectomy or radical trachelectomy. Median age was 38 years (range: 25–71.2) with 3 (21%) over age 50. All surgeries were elective, considered by definition complex and all but 1 (7%) had lymph node assessment performed. This patient having a stage IA1 cancer with negligible risk of nodal spread. Vertical midline incisions accounted for 86% of the incisions.

All patients were considered to be fully active and assigned a “0” performance status and 75% patients had an ASA score of 1, and 25% patient had an ASA of 2. Median body mass index (BMI) was 23.4 (range: 18.6–30.8) with 4 (29%) considered overweight and only 1 (7%) classified as obese.

Median operating time was 3.5 hours (range: 2.5–4.6). No patients had operations that lasted 2 hours or less, 4 (29%) had surgery durations between 2-3 hours, and 8 (57%) had surgeries lasting 3-4 hours. Two patients (14%) had surgeries lasting longer than 4 hours. Median EBL at surgery was 250 mL (range: 100–650) and median Hb change (preop to postop) was 13.5 g/L.

Median LOS was 3 days (range: 3–10) with average LOS of 3.9 days. No patients were discharged on day 2 and 1 (7%) patients had a LOS of greater than 7 days.

All patients tolerated EOF, there were no blood transfusions, readmissions, bladder, ureteric, bowel or vascular injury, no return to the operating room and no VTE diagnosed (Table 3).

### 13.1. Comment

FTS in patients undergoing either radical hysterectomy of radical abdominal trachelectomy is very well tolerated, with little or no major complications recorded in particular ureteric injury and readmission.

## 14. Day 2 or Super Early D/C

The improvement in surgical outcomes demonstrated in FTS programs has allowed as a consequence a reduction in the hospital LOS. We have previously reported that with experience, 1 in 3 patients undergoing a laparotomy for gynaecological surgery can be discharged on day 2 post surgery, without an increased morbidity or readmission rate [17, 94, 104].

Whilst it is probably unrealistic to expect the LOS after laparotomy to be further reduced from 2 days, it is realistic to expect that with further refinements and enhancements to FTS programs a greater proportion of patients can be safely discharge on day 2.

During the audit period 110 (28.3%) patients were discharged on day 2 after major abdominal surgery. Median age was 49.5 years (range: 20.1–79) with 55 (50%) under age 50, 39 (35%) between 50 and 65, 15 (14%) between 65 and 75 and 1 patient was older than age 75. Forty two (38%) had confirmed malignancy. Forty four percent of patients had prior abdominal surgery and the most common site of pathology was ovarian (52%), uterine corpus (34%) and uterine cervix (13%). Overall 68 (62%) had benign pathology and of those with malignant disease, 25% had stage I disease, 18% had stage II disease, 8% had stage III disease, and 4% had stage IV disease.

Surgery was considered complex in 93 (85%) patients and all considered elective. Lymph node sampling or dissection performed in 15 (14%). Vertical midline incisions accounted for 86 (78%) of the incisions.

Ninety two (84%) patients were considered to be fully active and assigned a “0” performance status, whilst 14 (13%) had light restrictions and assigned a performance status of “1”. Four (4%) were assigned a performance status of 2, not being able to work and being ambulatory 50% of the time. Fifty four percent of patients had an ASA score of 1, 25% had an ASA of 2 and 21%.

Median body mass index (BMI) was 25.9 (range: 17.5–44.5) with 30 (27%) considered overweight and 29 (26%) classified as obese.

Overall 110 (28.3%) of patients were discharged on day 2. In year 1, 7 of 73 (10%) of patients were discharged on day 2, in year 2 increasing to 25 of 99 (25%), 26 of 79 (33%) in year 3, 37 of 96 (39%) in year 4 and for 6 months of year 5, 15 of 42 (36%) were discharged on or before day 2 (Table 4).

Median operating time was 1.95 hours (range: 0.75–3.8). Three patients (3%) had operations that lasted less than 1 hour, 63 (57%) lasting between 1-2 hours, 38 (35%) had surgery durations between 2-3 hours, and 6 (5%) had surgeries lasting 3-4 hours. Median EBL at surgery was 150 mL (range: 10–900) and median Hb change (preop to postop) was 10 g/L.

All surgeries were considered “clean contaminated,” no patients received an intraoperative transfusion, 1 received a preop transfusion for menorrhagia and 1 received a transfusion postop after resection of a 20-week size fibroid uterus. All patients tolerated EOF. Wound infection occurred in 3 (3%) patients, 2 of whom were readmitted (2%) and 1 (0.9%) required return to the operating room for wound debridement. There was 1 (0.9%) bladder injury in a patient having had 2 prior caesarian sections, and 2 patients (2%) were readmitted, both with wound infections. There was 1 (0.9%) VTE found on a postop CT scan of chest (Table 3).

### 14.1. Comment

With increasing experience and the appointment of a dedicated fast track clinical nurse consultant (CNC), we have been able to increase the percentage of patients discharged on day 2 from 10% in the first year of the program to 25% in the second year and 33% in the third year, 39% in year 4, and currently in year 5 36% were discharged on day 2 after initiating a FTS program.

These improvements do not appear to be restricted to simple surgical cases in thin women, who have had transverse incisions and who lack private medical insurance. To the contrary, our data shows that 54% patients discharged on day 2 were considered overweight or obese, the majority (92%) had complex procedures performed, with 29% having lymph node assessment. That the majority (95%) had VMI and 34% having previous intra-abdominal surgery.

## 15. Obese Patients

It is an unfortunate reflection upon our society that the majority of Australians are considered overweight or obese and much of our nutrition is derived from supermarkets in prepackaged containers; that many individuals do not exercise, often lead a sedentary lifestyle and are more likely to drive a car down the street to run an errand rather than walk. The health implications of this “obesity epidemic” are enormous with direct and indirect consequences including type 2 diabetes, cardiovascular disease, sleep apnea, a higher risk of developing of some cancers, hypertension, and muscular-skeletal issues [105].

Obesity is most commonly measured using the body mass index (BMI), which is a weight to height ratio and is calculated by dividing the body weight in kilograms by the square of the height in metres (kg/m^2^). Adults with a BMI of greater than 25 are considered overweight and a BMI of greater than 30 is considered obese [106]. 

During the audit period, 121 (31%) of the group were classified as obese with a BMI of 30 or greater. Median age was 57 years (range: 25–85) with 68 (56%) having confirmed malignancy. Forty six percent of patients had prior abdominal surgery and the most common site of pathology was uterine corpus (52%), ovarian (45%) and uterine cervix (2.5%). Overall 53 (44%) had benign pathology and of those with malignant disease, 36% had stage I disease, 5% had stage II disease, 12% had stage III disease, and 2.5% had stage IV disease.

Surgery was considered complex in 112 (93%) patients and 94% were considered elective. Lymph node sampling or dissection performed in 16 (13%) and vertical midline incisions accounted for 99% of the incisions.

Fifty five (45%) patients were considered to be fully active and assigned a “0” performance status, whilst 47 (39%) had light restrictions and assigned a performance status of “1.” Seventeen (14%) were assigned a performance status of 2, not being able to work and being ambulatory 50% of the time whilst 2 (1.7%) patients had limited self care and were assigned a performance status of “3”. Twenty three percent 23% patients had an ASA score of 1, 44% had an ASA of 2 and 31% had an ASA of 3. One patient had an ASA of 4. Median body mass index (BMI) was 34.9 (range: 30.1–68.8) with a mean BMI of 36.5.

Median operating time was 2.3 (range: 1–5). Thirty four patients (28%) had operations that lasted 2 hours or less, 71 (59%) had surgery durations between 2-3 hours, and 13 (11%) had surgeries lasting 3-4 hours. Three patients (2.5%) had surgeries lasting longer than 4 hours. One (0.8%) patient had a formal radical trachelectomy and lymph node dissection performed for an early cervical cancer. Median EBL at surgery was 200 mL (range: 10–1000) and median Hb change (preop to postop) was 11 g/L.

Median LOS was 3 days (range: 2–16) with average LOS of 3.7 days. Overall 29 (24%) patients were discharged on day 2. A further 7 patients were suitable to be discharged on day 2 but for social reasons were unable to be discharged. 6 (5%) patients had a LOS of greater than 7 days.

One patient received an intraoperative transfusion, 2 (2.7%) did not tolerate EOF, 6 (5%) experienced a wound infection, 1 febrile morbidity, 2 (1.7%) bladder injuries, 7 (5.8%) hospital readmission, 3 (2.5%) VTE, 3 (7.5%) overall received a blood transfusion, 2 of which were preoperative. One patient was returned to the operating room and 2 (1.7%) had ICU admissions (Table 3).

### 15.1. Comment

While surgery remains the cornerstone of treatment for most gynaecologic cancers, obese, and overweight patients pose additional and increased challenges making the surgery generally technically more difficult, which often necessitates a modification of standard surgical treatment [107, 108]. This review has demonstrated that in overweight and obese women undergoing either complex gynaecological surgery or gynaeoncology surgery that outcomes are comparable to those of normal BMI. Obese and overweight patients are more likely to have a poorer performance status, have a greater likelihood of having a vertical midline incision than a transverse incision and due to associated comorbidities, are less likely to receive intraoperative or postoperative COX inhibitors for analgesia. It would appear that from our experience that patients classified as either overweight or obese undergoing a FTS protocol with an experienced surgeon, have similar outcomes after laparotomy when compared to patients of normal BMI. Surgery is more challenging but equally good outcomes can be achieved with a FTS or ESR program in overweight and obese patients having gynaecological surgery.

## 16. Elderly Patients

Gynaecological cancers are predominantly a disease of older women and with the elderly population growing at an ever increasing rate, more elderly women will be presenting for surgery. Whilst age appears to be an independent risk factor for morbidity and mortality [109], medical comorbidities are confounding variables. Indeed it is often not possible to separate age from comorbidities when deciding upon surgical options in the elderly [110].

To review the outcome of older and elderly patients undergoing a FTS program, patients aged 75 years and older were reviewed. Twenty three patients were considered elderly being 75 or greater at the time of their surgery. Median age in this subgroup of elderly patients was 80 years (range: 75–86) with 20 (87%) patients having confirmed malignancy. Eleven percent of patients had prior abdominal surgery and the most common site of pathology was uterine corpus (57%) and ovary (43%). Of those with malignant disease, 13 (57%) had stage I disease, 1 (4%) had stage II disease, 3 (22%) had stage III disease, and 1 (4%) had stage IV disease.

Surgery was considered complex in 22 (96%) patients and all surgeries considered elective. Lymph node sampling or dissection was performed in 5 (22%). Vertical midline incisions accounted for all incisions.

Two (9%) patients were considered to be fully active and assigned a “0” performance status, whilst 15 (65%) had light restrictions and assigned a performance status of “1.” Six (2.6%) were assigned a performance status of 2, not being able to work and being ambulatory 50% of the time. No patients had an ASA score of 1, 67% had an ASA of 2 and 33% had an ASA of 3. Median body mass index (BMI) was 27.3 (range: 20.6–40.1) with 12 (52%) considered overweight and 7 (30%) classified as obese.

Median operating time was 2 hours (range: 1–3.5). 12 patients (52%) had operations that lasted 2 hours or less, 9 (39%) had surgery durations between 2-3 hours, and 2 (9%) had surgeries lasting 3-4 hours. Median EBL at surgery was 150 mL (range: 50–1000) and median Hb change (preop to postop) was 7 g/L.

Median LOS was 4 days (range: 2–13) with average LOS of 4.8 days. Overall only 1 (4%) patient was discharged on day 2. A further 1 patient was suitable to be discharged on day 2 but for social reasons were unable to be discharged. Three (13%) patients had a LOS of greater than 7 days.

One (4%) patient experienced a wound infection, 1 (4%) patient did not tolerate EOF, there were 2 (95) patients readmitted, 1 (4%) was diagnosed with VTE, 1 (4%) had an ICU admission, and 1 (4%) patient received a blood transfusion (Table 3).

### 16.1. Comment

Even though elderly patients are at an increased risk of development of gynaecological cancers and are more likely to die of their disease, they often receive less aggressive surgery, less likely to receive adjuvant therapy and when offered that adjuvant therapy may be dose reduced compared to younger patients. Even after medical comorbidities are considered, elderly patients aged over 80 have increased morbidity and mortality [109, 111].

This audit has shown that elderly patients generally have a poorer performance status and ASA score and are likely to be obese. Surgery was undertaken in a relatively reasonable time with minimal blood loss. Whilst tolerating an FTS program, elderly patients spend longer in hospital compared to their younger counterparts, but their complication rate is not substantially increased. 

## 17. Conclusion

 The data from this rolling clinical audit, representing the largest of its kind reported, confirms the overall safety of fast-track surgery in patients undergoing surgery for suspected gynaecological cancer.

## Figures and Tables

**Table 1:** Quality indicators for all patients on FTS program: January 2008–June 2012.

	*N* (%)
Wound dehiscence	0
Wound infection	10 (2.6%)
Febrile morbidity	3 (0.8%)
Ureteric injury	0
Bladder injury	2 (0.5%)
Bowel injury	0
Vascular injury	0
VTE	4 (1%)
Transfusion	8 (7%)
Preop transfusion	3 (2.7%)
Transfusion >2 units	4 (1%)
Anastomotic leak	0
Return to OR	2 (0.5%)
Death <30 days post op	0
Undiagnosed cancer	0
ICU admission	4 (1%)

**Table 2:** Univariate and multivariate analysis of variables associated with reduced length of stay.

Univariate analysis
Tumour site (cervix)
Benign pathology
Early stage
Local versus distant
<50 versus 65 or greater
<50 versus 75 or greater
50 greater versus 75 or greater
Private
VMI versus transverse
Year 4, 5 versus 1
ASA 1 versus 3
Surgery duration
Elective
No intra-op transfusion
COX Inhibitor
Zero performance status
EOF
Wound infection
Febrile morbidity
Bladder injury
Readmission
VTE
Return OR
ICU admit
Younger age
Shorter operating time
Increasing height
No, low units transfused
Low EBL
Increased pre-op Hb
Increased post-op Hb
Low net Hb change

Multivariate analysis

Benign pathology
Shorter operating time
Tolerated EOF

**Table 3:** Summary data of all patients and subgroup analysis after surgery implementation of an FTS program.

	All	Benign	Ovarian cancer	Uterine cancer	Cervical cancer	Obese	Elderly	Radical Hyst	Day 2
Age (yrs)	55	49	57.3	60.5	40.5	57	80	40	49.5
Complex	90%	78%	99%	98%	95%	93%	96%	100%	85%
Nonzero PS	32%	19%	42%	50%	0	54%	91%	0	16%
VMI	90%	82%	98%	99%	68%	99%	100%	89%	78%
Weight (kg)	68	69	65	77	56	91	67	62	66
BMI	26.5	26.5	24.6	28.8	22.4	34.9	27.3	23.6	25.9
Obese	31%	33%	17%	44%	14%	100%	30%	5%	26%
OR Time (hrs)	2.25	2	2.5	2.3	3	2.3	2	3.5	1.95
EBL (mL)	175	150	250	150	150	200	150	250	150
Hb change (g/L)	11	12	11	9.5	10.5	11	7	12	10
LOS (days)	3	3	4	3	3	3	4	3	2
EOF	95%	95%	85%	100%	100%	97%	92%	100%	100%
Readmission rate	4%	2.5%	5.6%	6.3%	0	5.8%	8.7%	5%	1.8%
Return to OR	0.5%	0	0	1.8%	0	0.8%	0	0	0.9%
Wound infection	2.6%	1.9	1.1%	4.5%	0	5%	4%	0	2.7%
Ureteric injury	0	0	0	0	0	0	0	0	0
Bladder injury	0.5%	0.6%	0	0.9%	0	1.7%	0	0	0.9%
Bowel injury	0	0	0	0	0	0	0	0	0
VTE	1%	2.5%	1.1%	1.8%	0	2.5%	4.3%	0	0.9%
IntraopTx	2.8%	1.9%	7.8%	0	0	0.8%	4.3%	5%	0

**Table 4:** Incidence of discharge on day 2 after laparotomy compared with year of surgery after initiation of FTS program.

	Year 1	Year 2	Year 3	Year 4	Year 5
D/C day 2	10%	25%	33%	39%	36%
